# Assessing the severity of pulmonary embolism among patients in the emergency department: Utility of RV/LV diameter ratio

**DOI:** 10.1371/journal.pone.0242340

**Published:** 2020-11-19

**Authors:** Sung-uk Cho, Young-duck Cho, Sung-hyuk Choi, Young-hoon Yoon, Jong-hak Park, Sung-joon Park, Eu-sun Lee

**Affiliations:** 1 Department of Emergency Medicine, Korea University Guro Hospital, Seoul, Korea; 2 Department of Emergency Medicine, Korea University Ansan Hospital, Ansan, Korea; Azienda Ospedaliero Universitaria Careggi, ITALY

## Abstract

**Background:**

Acute pulmonary embolism (APE) is a major cause of death from cardiovascular disease. Right ventricular systolic dysfunction (RVD) caused by APE is closely related to a poor outcome. Early risk stratification of APE is a vital step in prognostic assessment. The objective of this study was to investigate the usefulness of computed tomographic pulmonary angiography (CTPA) measured right ventricular (RV)/ left ventricular (LV) diameter ratio by the emergency department (ED) specialists for early risk stratification of APE patients in ED.

**Methods:**

The retrospective data of 229 APE patients were reviewed. Two ED specialists measured both RV and LV diameters on a single transverse scan perpendicular to the long axis of the heart. The patients were divided into two groups, RV/LV diameter ratio <1 and ratio >1. CTPA measured RV/LV diameter ratio were analyzed and compared with sPESI score, cardiac biomarkers such as N-Terminal Pro-B-Type Natriuretic Peptide (NT-pro-BNP), high sensitivity cardiac troponin T (hs-cTnT), and RVD measured by echocardiography (Echo).

**Results:**

The mean age in RV/LV > 1 group was significantly higher than that of the other group (67.81±2.7 years vs. 60.68±3.2 years). Also, there were more hypertension patients (44.4% vs. 33.3%), and mean arterial pressure (MAP) was lower. A significantly higher ICU admission rate (28.05% vs. 11.61%) was shown in RV/LV >1 group, and five patients expired only in RV/LV > 1 group. RVD by Echo demonstrated the highest sensitivity, specificity, and negative predictive value (NPV) (values of 94.3%, 81.1%, 95.5%). RV/LV >1 diameter ratio by CTPA showed usefulness equivalent to cardiac biomarkers. RV/LV >1 patients’ cardiac enzymes were higher, and there were more RVD in RV/LV >1 group.

**Conclusion:**

Simple measurement of RV/LV diameter ratio by ED specialist would be a help to the clinicians in identifying and stratifying the risk of the APE patients presenting in the ED.

## Introduction

Acute pulmonary embolism (APE) is known as the third leading cause of death from cardiovascular disease [1]. The increased use of thrombolytic therapy and surgical embolectomy has led to a significant improvement in the prognosis of APE patients over the past decades. The results obtained from a European registry of 23,000 patients followed over 12 years showed a decrease in the all-cause mortality at 30 days, from 3.3% to 1.38% [2]. Nevertheless, the risk stratification of APE patients in the emergency department (ED) remains a challenge to physicians.

APE has a highly variable clinical course ranging from asymptomatic presentation to hemodynamic instability, right heart dysfunction, and failure, eventually leading to death [3, 4]. Massive APE patients, defined by systemic hypotension, should be considered a high-risk group, and vital therapeutic options such as thrombolysis or embolectomy need to be done [5, 6].

Early risk stratification of APE is considered a vital step in facilitating prognosis assessment and guiding therapeutic decision-making [4]. Previous studies have shown that cardiac biomarkers such as cardiac troponin and N-terminal pro-brain type natriuretic peptide (NT-pro-BNP) are effective in identifying right ventricular systolic dysfunction (RVD). Also, elevated cardiac biomarkers are closely associated with high mortality in APE patients [7, 8].

The diagnosis of PE in the ED is based on clinical probability evaluation, laboratory testing, echocardiography, and computed tomographic pulmonary angiography (CTPA) [9]. CTPA has been recommended as a first-line examination for the diagnosis of APE [4, 10].

The scoring system has been adapted for severity assessment. The pulmonary embolism severity index (PESI) score, which was introduced in 2005, had 11 routinely available clinical predictor variables with prognostic values. Based on the PESI score, the patients are categorized into five classes with 30-day mortality ranging from 1.1% to 24.5% [11, 12]. In 2010, the simplified PESI (sPESI) was developed with six of the 11 original PESI variables [13]. It was used to categorize patients into two classes: low risk, with none of the variables, and high risk, including one to six variables.

The objective of this study was to investigate the usefulness of CTPA measured right ventricular (RV)/ left ventricular (LV) diameter ratio by the ED specialists for early risk stratification of APE patients (predicting the intensive care unit (ICU) admission and in-hospital mortality) in the ED. Also, sPESI score, cardiac biomarkers such as N-Terminal Pro-B-Type Natriuretic Peptide (NT-pro-BNP), high sensitivity cardiac troponin T (hs-cTnT), RVD measured by echocardiography (Echo) were analyzed for comparison and assessment.

## Materials and methods

Korea University Guro Hospital Institutional Review Board (no.2020GR0211) approved this study and waived the requirement for written informed consent because the analysis used anonymized data, which were obtained retrospectively.

This retrospective cohort study entailed a review of the electronic medical records of 314 APE patients who visited the ED from 2014 to 2017 at two tertiary hospitals located in Korea.

The charts were reviewed by two ED specialists. When conflicting data for continuous variables were presented to the chart reviewers, they used the mean value of the above-mentioned data. When the categorical variables were different, the third researcher decided which results to use after reviewing the chart.

Patients with symptoms pathognomonic for PE, such as chest pain, dyspnea, hemoptysis, and syncope, were diagnosed by the presence of thromoboemboli in at least one segmental pulmonary artery on contrast-enhanced multi-detector computed tomography.

Commercial echocardiography (Vivid E9, Vivid 7 GE Vinged Ultrasound AS, Horten, Norway) was used for evaluation of heart function. Complete two-dimensional, color flow, pulsed-wave, and continuous-wave Doppler examinations were performed according to standard techniques. RV free wall hypokinesia or akinesia demonstration by echocardiography was considered as definite evidence of RVD.

**Fig 1:**
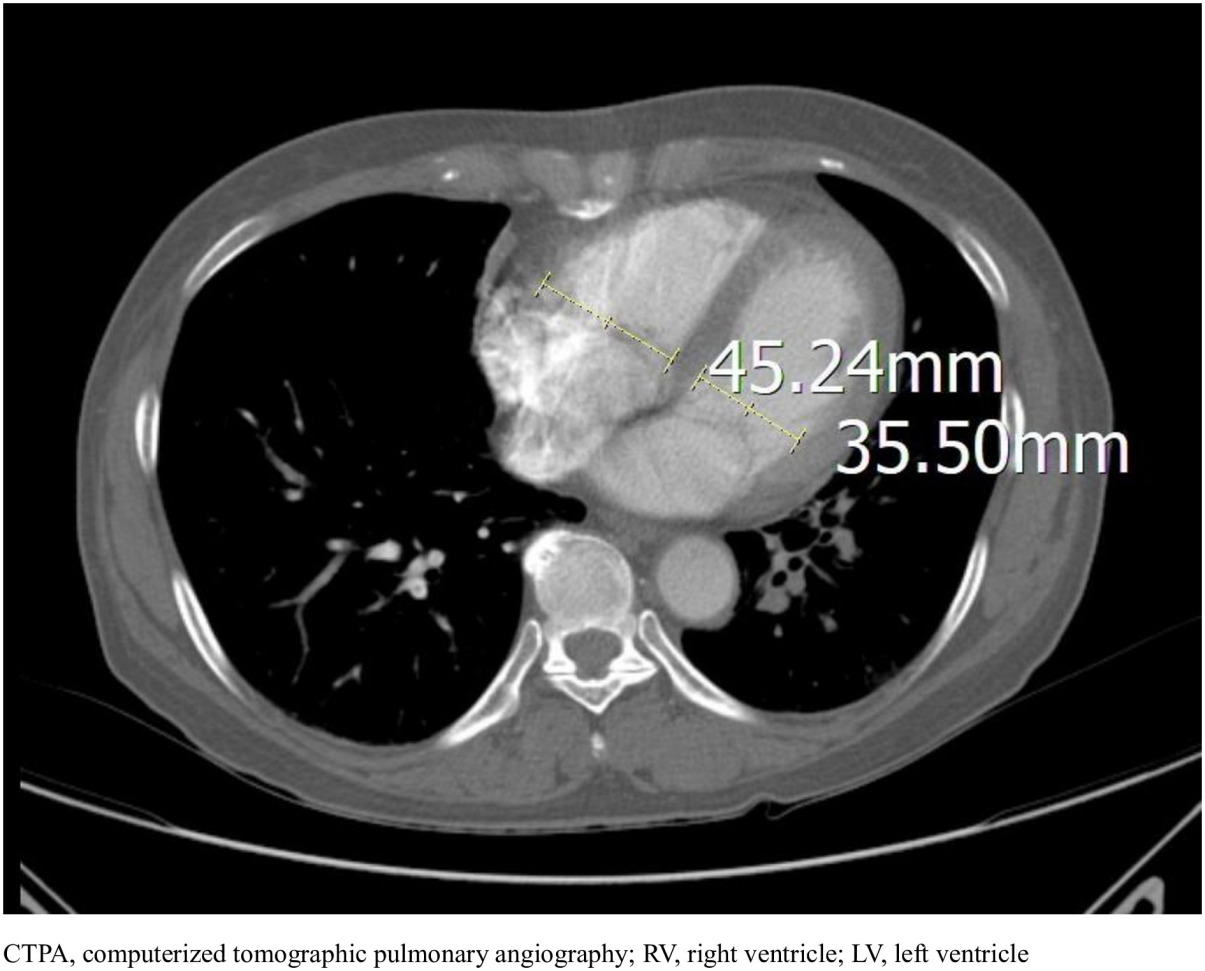
CTPA illustration of RV/LV diameter ratio measurement: Measurement in this patient was 1.28.

All CTPA examinations were conducted with the Siemens SOMATOM Definition edge device (Siemens Aktiengesellschaft, Munich, Germany). All CTPA images were retrospectively reviewed by 2 ED specialists. They were blinded to the clinical information, including treatments and outcomes. They measured both RV and LV diameters on a single transverse scan perpendicular to the long axis of the heart. Its definition was the largest distance between the inner aspect of the interventricular septum and the ventricular free wall (Fig 1).

NT-pro-BNP was measured with a fluorescence immunoassay using a cobas^®^ 8000 modular analyzers (Roche Diagnostics, Mannheim, Germany). The detection limit was 18 ng/mL, and the measurement range was 18–35,000 ng/mL. NT-pro-BNP cut-off values were used, as illustrated in the textbook [14].

According to the European Society of Cardiology, hemodynamic instability was defined by a systolic blood pressure below 90 mm Hg or a need for vasopressor without other causes such as hypovolemia or septic shock condition [15].

Parameters such as NT-pro-BNP, hs-cTnT, RVD, and the RV/LV ratio via CTPA were investigated to evaluate the correlation between cardiac function and severity of APE. NT-pro-BNP levels were marked as elevated based on NT-pro-BNP cut-off values. Further, the RV and left ventricle (LV) diameter ratio were recorded based on CTPA results of RV function evaluation.

In predicting the possibility of ICU admission, sensitivity, specificity, positive predictive value (PPV), and negative predictive value (NPV) were calculated from RV/LV diameter ratio, elevated cardiac biomarkers, and RVD by Echo.

We used SPSS software (SPSS 21.0, IBM, Chicago, USA) for statistical analysis. A *P* value of < 0.05 was considered to show statistical significance. Each continuous variable was expressed as a mean ± standard deviation (SD).

We conducted an ANOVA or Kruskal-Wallis test to compare the mean value in groups depending on the normality test. A Fisher exact test was used to evaluate the correlation of each. Spearman’s correlation coefficient was used to measure the degree of association between groups.

Early risk stratification time cut-off was immediately after CTPA was taken. The risk stratification needed to be done before Echo was done. The sample size of this study was calculated with the intention to conduct a prospective evaluation for future study.

## Results

**Fig 2:**
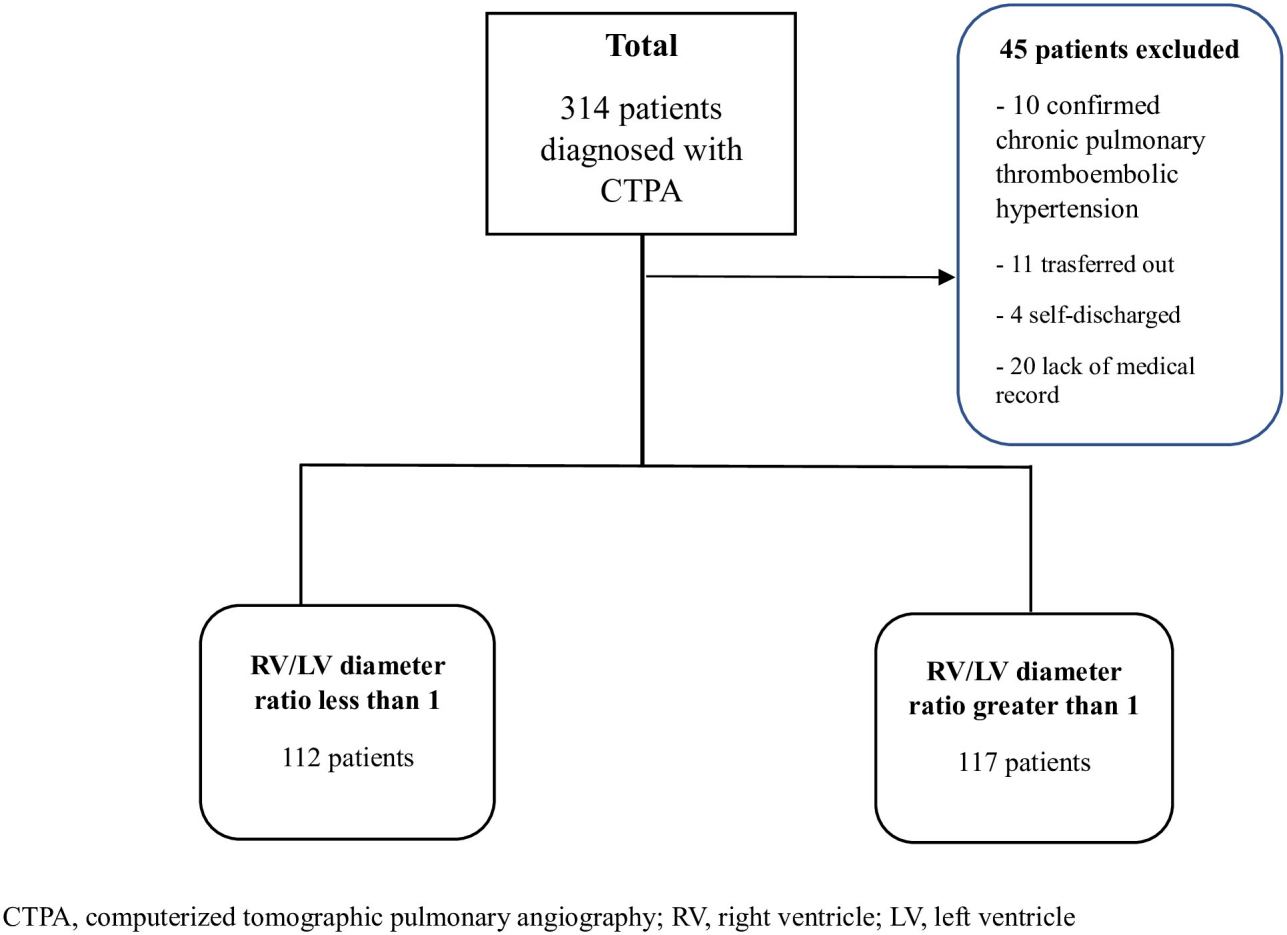
Flowchart of the study selection process. Risk stratification according to RV/LV diameter ratio.

Between January 2014 and December 2017, 314 patients were diagnosed with APE using CTPA in the ED and 45 patients with confirmed chronic hypertension related to pulmonary thromboembolism, those transferred out of hospitals, self-discharged, or without medical records were excluded. Ultimately, 229 patients were enrolled. Patients were divided into two groups according to the RV/LV diameter ratio by CTPA (Fig 2).

### Demographic and clinical characteristics of acute pulmonary embolism patients in different RV/LV ratio groups

The mean ages of each group were 60.68±3.2 years for RV/LV diameter ratio < 1 group and 67.81±2.7 years for RV/LV diameter ratio >1 group (*p* < 0.001). There were more males (49.1% vs. 38.5%), and diabetes mellitus (DM) patients (32.1% vs. 24.8%) in RV/LV diameter ratio <1 group. There were more patients with systemic arterial hypertension (44.4% vs. 33.3%) in RV/LV diameter ratio >1 group. Mean arterial pressure (MAP) was recorded higher in RV/LV diameter ratio <1 group, and the mean heart rate (HR) was faster in RV/LV diameter ratio > 1 group (*p* < 0.001). In both groups, dyspnea and chest pain were the two most common symptoms (Table 1).

**Table 1:** Demographic and clinical characteristics of acute pulmonary embolism patients in different RV/LV diameter ratio groups.

	RV/LV <1(n = 112)	RV/LV >1 (n = 117)	P-value
Age (years, mean ± SD)	60.68±3.2	67.81±2.7	< 0.001
Male, n (%)	55 (49.1)	45 (38.5)	0.107
HTN, n (%)	37 (33.3)	52 (44.4)	0.276
DM, n (%)	36 (32.1)	29 (24.8)	0.222
MAP (mmHg, mean ± SD)	100.04±2.8	92.96±3.3	< 0.001
HR (min, mean ± SD)	90.46±3.2	98.39±3.3	< 0.001
Symptoms, n (%)			
Hemoptysis	7 (6.3)	7 (5.9)	0.899
Dyspnea	58 (51.8)	80 (68.4)	0.01
Chest pain	13 (11.6)	24 (20.5)	0.067
Syncope	4 (3.6)	3 (2.6)	0.663
Lower extremity swelling	5 (4.5)	3 (2.6)	0.437
Lower extremity pain	10 (10.7)	1 (0.8)	0.001
Abdomen pain	8 (7.1)	7 (5.9)	0.713
Miscellaneous	20 (17.9)	5 (0.4)	11.61

SD, standard deviation; MAP, mean arterial pressure; HR, heart rate; DM, diabetes mellitus; HTN hypertension; RV, right ventricle; LV, left ventricle.

### ICU admission and mortality in each RV/LV ratio group

Significantly higher ICU admission rate (28.05% vs. 11.61%) was shown in RV/LV diameter ratio >1 group (*p*<0.001) and five patients expired only in RV/LV diameter ratio > 1 group (*p*<0.001) (Table 2).

**Table 2:** ICU admission and expired patients in each RV/LV diameter ratio groups.

	RV/LV <1 (n = 112)	RV/LV >1 (n = 117)	P-value
ICU admission (n, %)	13 (11.61)	54 (28.05)	< 0.001
Expired (n, %)	0 (0)	5 (4.3)	0.01

ICU, intensive care unit. RV, right ventricle; LV, left ventricle.

### Performance of RV/LV diameter ratio >1, elevated NT-pro-BNP, RVD detected by Echo, and elevated hs-cTnT for predicting ICU admission

RVD by Echo demonstrated the highest sensitivity, specificity and NPV (values of 94.3, 81.1, 95.5). RV/LV >1 diameter ratio by CTPA showed usefulness equivalent to cardiac biomarkers (NT-pro-BNP, hs-cTnT) (*p* < 0.001) (Table 3).

**Table 3:** Performance of RV/LV diameter >1, elevated NT-pro-BNP, RVD detection by Echo, and elevated troponin for predicting ICU admission.

	Sensitivity	Specificity	PPV	NPV	P-value
RV/LV > 1 (95% CI)	80.6 (69.11 ~ 89.24)	58.6 (50.29~66.48)	46.2 (40.69~51.71)	87.3 (80.50~91.91)	<0.001
Elevated NT-pro-BNP (95% CI)	85.1 (74.7 ~ 91.7)	63.0 (55.3 ~ 70)	48.7 (39.8 ~ 57.7)	91.1 (84.3~95.1)	<0.001
RVD detected by Echo (95% CI)	94.3 (87.2~97.5)	81.1 (73.5~86.8)	76.6 (67.8~83.6)	95.5 (90.0~98.1)	<0.001
Elevated hs-cTnT (95% CI)	79.3 (61.6~90.2)	53.0 (46.1~59.8)	19.7 (13.5~27.8)	94.6 (88.8~97.5)	<0.001

NT-pro BNP, N-terminal pro b-type natriuretic peptide; RV, right ventricle; LV, left ventricle; Echo, echocardiography; hs-cTnT, high sensitivity cardiac troponin T; RVD, right ventricular systolic dysfunction; CI, confidence interval.

### Composition of patients in each parameter

**Fig 3:**
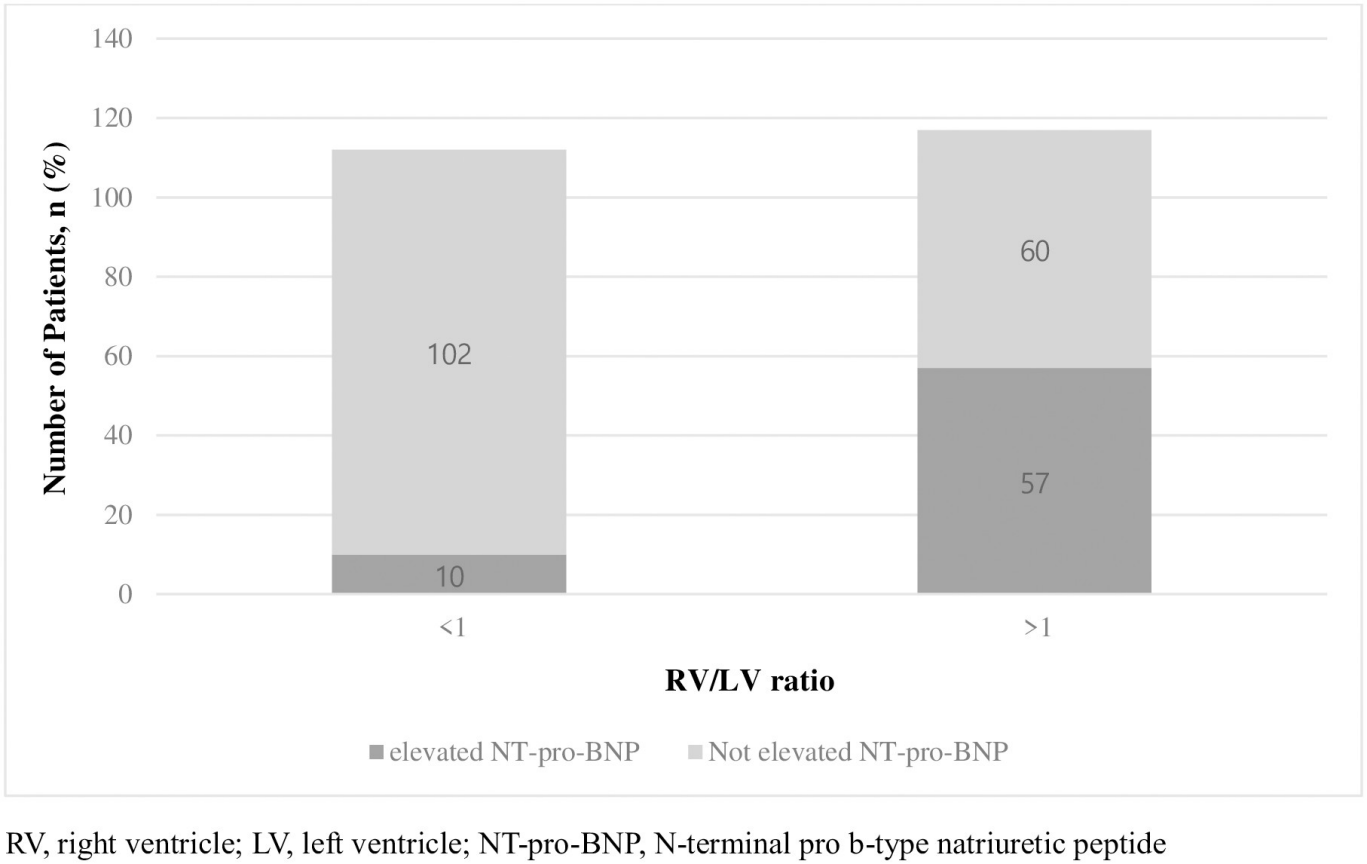
Composition of elevated and not elevated NT-pro-BNP patients in each RV/LV diameter ratio groups. (P = 0.000).

Ten patients (8.9%) with elevated NT-pro BNP were in RV/LV diameter ratio <1 group and 57 patients (48.7%) were with elevated NT-pro BNP in RV/LV diameter ratio >1 group (P<0.001) (Fig 3).

**Fig 4:**
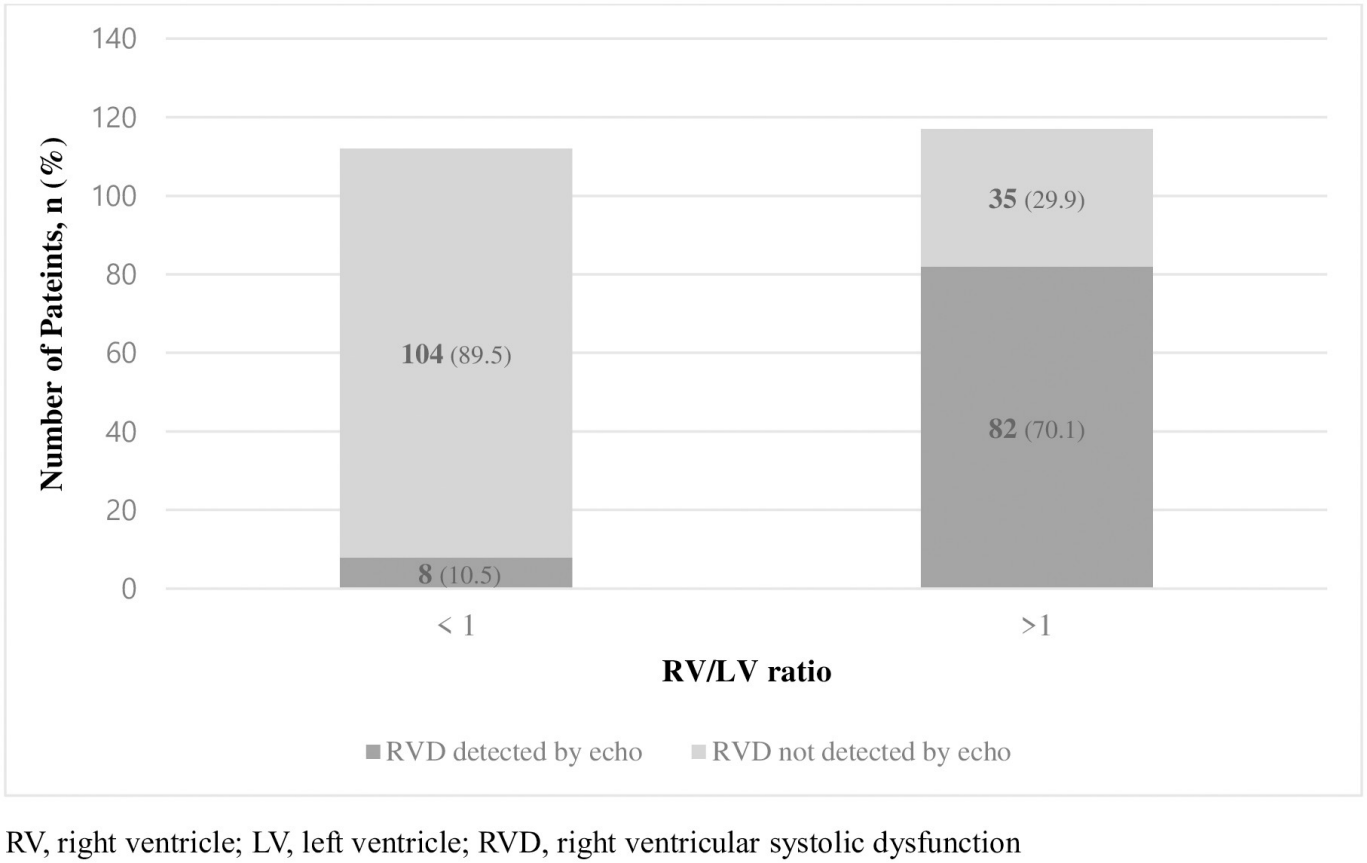
Composition of RVD detected by Echo and RVD not detected by Echo patients in each RV/LV diameter ratio groups. (P< 0.001).

**Fig 5:**
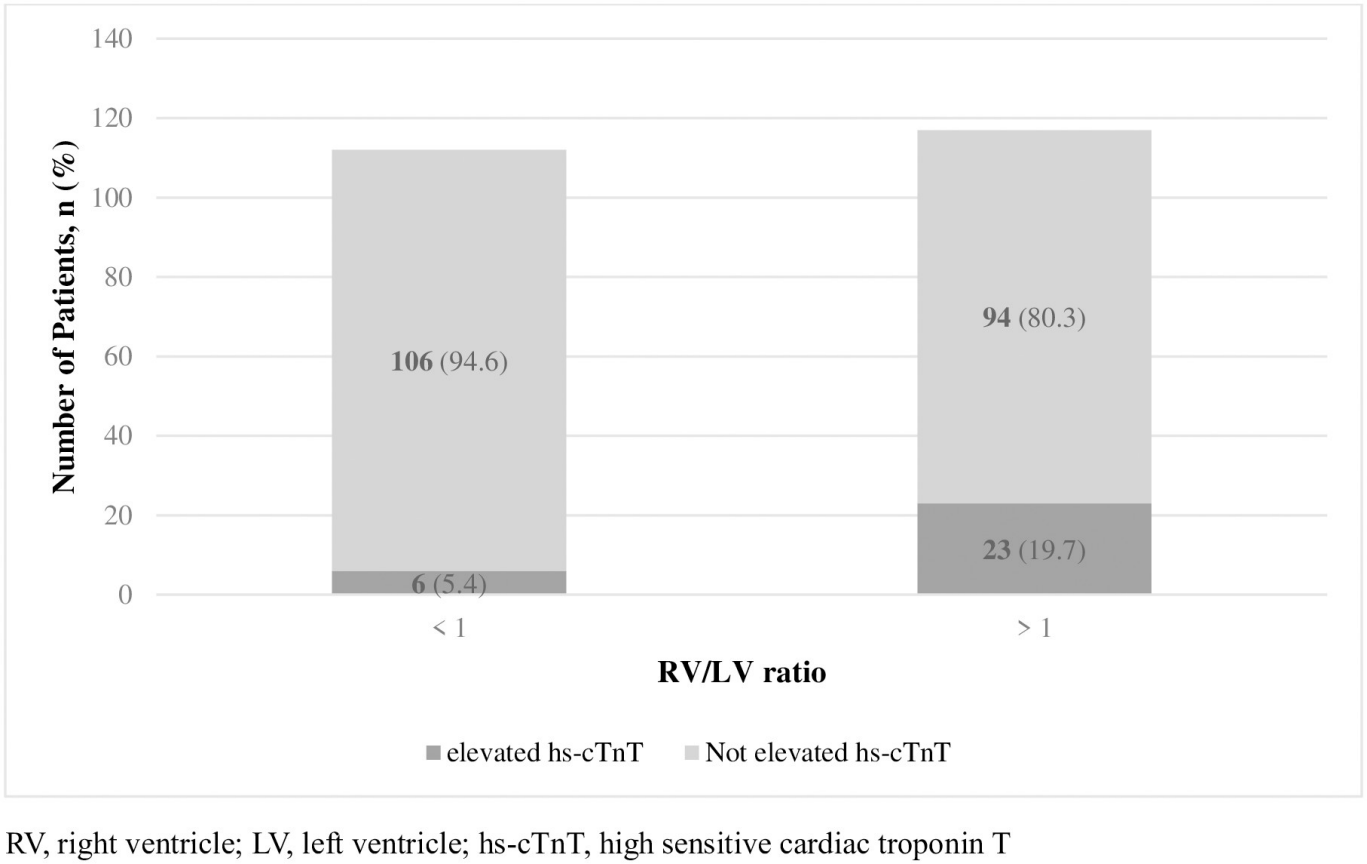
Composition of RVD detected by Echo and RVD not detected by Echo patients in each RV/LV diameter ratio groups. (P = 0.001).

There were 8 patients (10.5%) with RVD detected by Echo in RV/LV diameter ratio <1 group and 82 patients (70.1%) in >1 group (*p*<0.001) (Fig 4). Result of elevated hs-cTnT patients’ composition in RV/LV diameter ratio >1 group were similar. It showed 23 patients (19.7%) in RV/LV diameter ratio >1 group compared to 6 patients (5.4%) in RV/LV diameter ratio < 1 group (*p* = 0.001) (Fig 5).

### sPESI score distribution in each RV/LV ratio group

**Fig 6:**
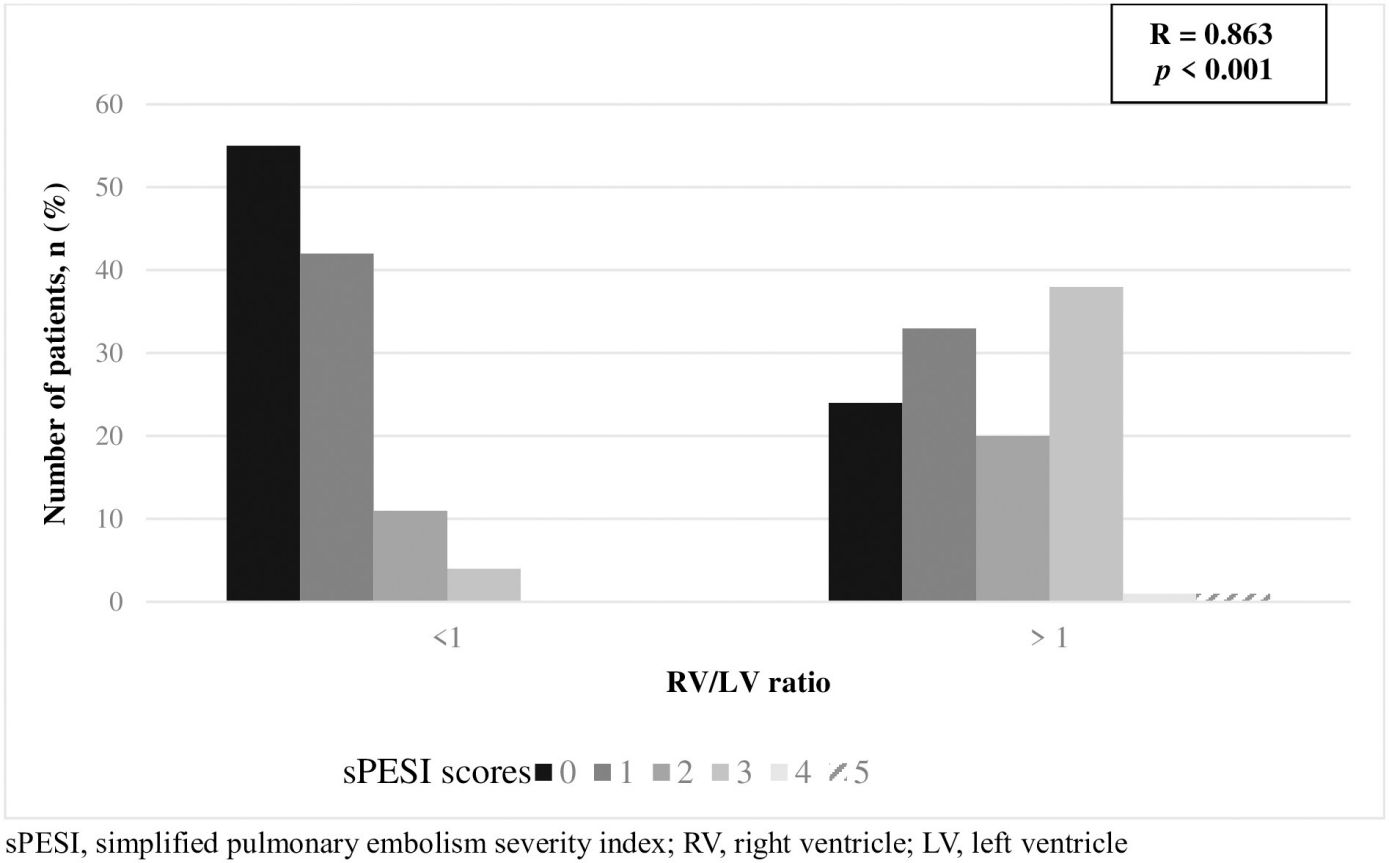
sPESI score distribution in each RV/LV diameter ratio groups.

In contrast to the RV/LV diameter ratio <1 group, RV/LV diameter ratio >1 group showed significantly more number of scores 2 and 3 patients. Also, there were not any score 4 and 5 patients in RV/LV diameter ratio <1 group, whereas RV/LV diameter ratio >1 group included 4 and 5 score patients, one each. sPESI score showed a significant positive correlation with RV/LV diameter ratio groups (R = 0.863, *p*<0.001) (Fig 6).

## Discussion

Several studies have shown reliable evidence suggesting that the prognosis of APE patients is influenced by hemodynamic status, right ventricular dysfunction with or without myocardial injury, and other clinical features [16, 17]. Based on our study results, older patients tend to manifest a higher risk than younger patients, and a lower MAP was observed among RV/LV diameter ratio >1 group. It is possibly attributed to underlying comorbidity in older patients, and groups with elevated risks exhibit unstable vital signs. In-hospital mortality and ICU admission rates were significantly higher in the RV/LV diameter ratio >1 group; there was no mortality in the RV/LVdiameter ratio >1 group. Furthermore, there seem to be strong correlations between RV/LV diameter ratio by CTPA and other parameters such as cardiac biomarkers, Echo findings, and sPESI scores in risk stratification. This study showed that CTPA is used not only as a diagnostic tool but also to assess the severity of APE and the RV function, as well as predict the prognosis such as ICU admission and mortality.

CTPA has been an effective tool in APE diagnosis as well as in the process of the clinical decision making of therapeutic strategy and risk stratification [18]. Echo can be used to evaluate the overall function of the heart, especially the assessment of the RV. It can be used to detect RVD and facilitate the diagnosis of APE as well as risk stratification [19, 20]. Nonetheless, the precise role of echocardiographic RVD in APE diagnosis or risk stratification has yet to be demonstrated [21]. RV dilation based on CTPA result as a measure of RVD has shown a strong correlation with echocardiographic parameter and to predict a higher 30–day mortality risk [22, 23].

RV/LV diameter ratio measurement on CTPA has an advantage over other tests such as Echo. It eliminates the unnecessary additional testing for RV evaluation in addition to the diagnostic testing for APE confirmation.

This study has a number of limitations. The main limitation is that this is not a randomized controlled study and may have a selection bias of subjects. Also, the timing of blood sampling from cardiac biomarkers may be different for each patient. And, other possible signs of RVD from CTPA were not considered. Finally, other echocardiographic measurements such as RV FAC, RV diameter, RA diameter, LV diameter, and TAPSE were not analyzed in this study. However, such parameters can be accepted with the intention of creating a hypothesis that would be analyzed prospectively in our next study.

The accuracy of RV/LV diameter ratio measurement on CTPA by non-radiologists has been studied, and the accuracy and reproducibility have been proven [24]. This is relevant for emergency medicine specialists in the ED. Most of the APE patients are initially diagnosed and risk assessed in the ED, and their outcomes and prognosis may depend on the initial assessment.

## Conclusion

Simple measurement of RV/LV diameter ratio by ED specialist would be a help to the clinicians in identifying and stratifying the risk of the APE patients presenting in the ED.

## Supporting information

S1 Data (XLSX)
